# Beta Modulation Depth Is Not Linked to Movement Features

**DOI:** 10.3389/fnbeh.2019.00049

**Published:** 2019-03-14

**Authors:** Elisa Tatti, Serena Ricci, Ramtin Mehraram, Nancy Lin, Shaina George, Aaron B. Nelson, Maria F. Ghilardi

**Affiliations:** ^1^CUNY School of Medicine, New York City, NY, United States; ^2^Dipartimento di Informatica, Bioingegneria, Robotica e Ingegneria dei Sistemi (DIBRIS), University of Genova, Genoa, Italy; ^3^Institute of Neuroscience, Newcastle University, Newcastle upon Tyne, United Kingdom

**Keywords:** EEG, human, motor control, velocity, ERD, ERS, movement length

## Abstract

Beta power over the sensorimotor areas starts decreasing just before movement execution (event-related desynchronization, ERD) and increases post-movement (event-related synchronization, ERS). In this study, we determined whether the magnitude of beta ERD, ERS and modulation depth are linked to movement characteristics, such as movement length and velocity. Brain activity was recorded with a 256-channels EEG system in 35 healthy subjects performing fast, uncorrected reaching movements to targets located at three distances. We found that the temporal profiles of velocity were bell-shaped and scaled to the appropriate target distance. However, the magnitude of beta ERD, ERS and modulation depth, as well as their timing, did not significantly change and were not related to movement features.

## Introduction

Voluntary movements are associated with EEG oscillatory activity in different frequency bands (Babiloni et al., 2016, 2017). In particular, the power of beta rhythm (15–30 Hz) recorded over sensorimotor areas decreases before movement onset, reaches its negative peak during execution (event-related desynchronization, ERD) and sharply rebounds afterwards (event-related synchronization, ERS; Pfurtscheller and Lopes da Silva, 1999; Toma et al., 2002; Kilavik et al., 2013). These movement-related changes can be captured by modulation depth, a measure independent from general spectrum changes (computed as the difference between maximal ERD and ERS) that increases with practice (Nelson et al., 2017). In general, beta activity reflects inhibition of the motor system, as it is associated with increased local GABAergic tone (Cassim et al., 2001; Gaetz et al., 2011; Muthukumaraswamy et al., 2013) and decreased cortical excitability (Hsu et al., 2011; Noh et al., 2012; McAllister et al., 2013). Hence, beta ERD may release motor areas from idle state to plan and execute movements, while beta ERS may reflect post-movement active inhibition of the motor network and reactivation of somatosensory areas (Alegre et al., 2008; Solis-Escalante et al., 2012). This oscillatory dynamic is present during movements with different effectors and characteristics (Pfurtscheller and Lopes da Silva, 1999; Tombini et al., 2009; Moisello et al., 2015; Nelson et al., 2017). However, some important questions still remain unanswered. For instance, do beta ERD and ERS magnitudes reflect specific motor characteristics? Or are they related to other factors? Indeed, many studies failed to find association between beta ERD or ERS magnitude and movement parameters such as speed (Stancák and Pfurtscheller, 1995, 1996), force (Pistohl et al., 2012; Cremoux et al., 2013) and muscle pattern (Salmelin et al., 1995). Yet, other studies found that beta ERS amplitude increased with increasing movement speed or force (Stančák et al., 1997; Parkes et al., 2006; Fry et al., 2016). Nevertheless, it is indisputable that beta modulation is linked to movement planning and execution.

One important aspect of efficient fast, uncorrected movements is the specification of an impulse force appropriate for that movement distance, thus with a force scaling factor applied to a set pattern of muscles (Ghez and Gordon, 1987). Ultimately, this results in scaled velocity temporal profile, so that movements to more distant targets have higher peak accelerations and velocities than those to closer targets (Gordon et al., 1994a,b). This phenomenon (“height control”) applies to multi-joint movements and can be accompanied by small but significant changes in movement time. Here, we used a task with such characteristics that allows for parameterization of peak force without changing muscle pattern and proprioceptive input: subjects were asked to reach targets at different distances with fast, uncorrected movements. These movements are associated with beta modulation changes (Moisello et al., 2015; Nelson et al., 2017). As per previous experiments, we expected that a stereotyped bell-shaped velocity profile is scaled to the appropriate target distance (Gordon et al., 1994a,b). Moreover, if the magnitude of beta ERD, ERS and modulation depth mostly reflected the planned impulse force, we expect that longer movements would correspond to greater beta modulation.

## Materials and Methods

### Subjects

Thirty-five healthy, right-handed subjects (age range: 19–35, mean ± SD: 24.1 ± 4.7 years, 19 female) participated in this study that was approved by CUNY Institutional Review Board (IRB). All subjects signed IRB-approved informed consent forms.

### Experimental Design

Subjects were fitted with a 256-electrode HydroCel Geodesic Sensor Net. They were comfortably seated in a sound-shielded room in front of a display; EEG activity was recorded while they performed a motor task with their right arm as detailed below.

### Motor Task

**Figure 1:**
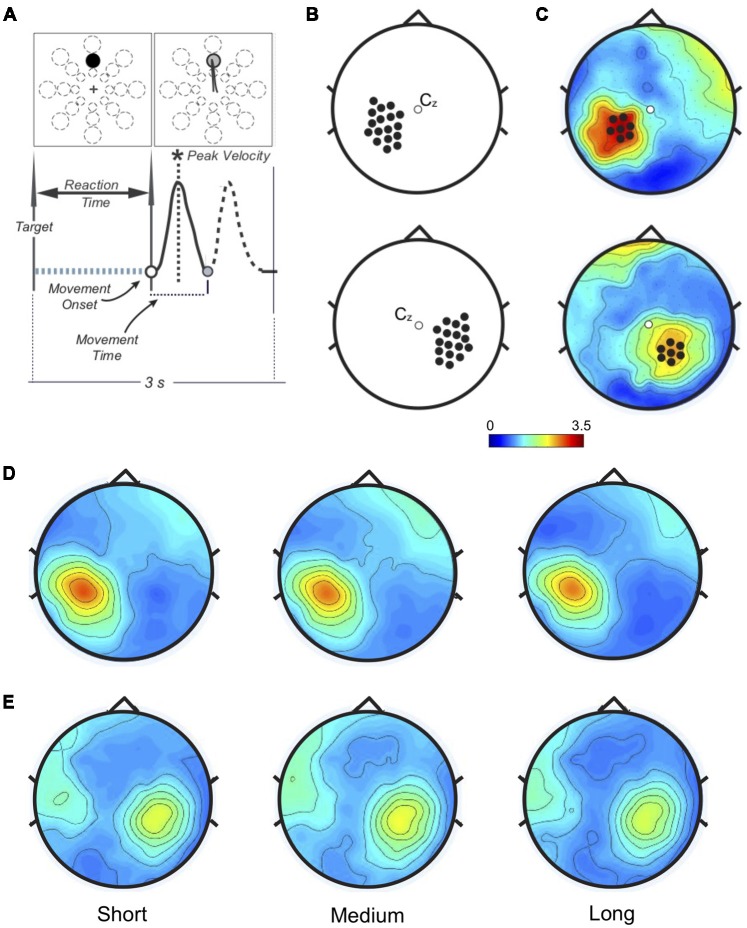
**(A)** Task and performance measures. Top: one of 24 targets (three distances, eight directions) appeared in unpredictable order every 3 s. Bottom: performance measures related to the movement. Only the forward movement (solid line) was considered for this study. **(B)** Left (top) and Right (bottom): sensorimotor areas. Beta modulation depth was computed for each electrode in each subject to find the peak value.** (C)** Example of personalized regions of interest (ROIs) in one subject. The peak electrode and the six neighbor ones for both Left (top) and Right (bottom) somatosensory regions define the personalized ROIs. **(D)** Left and** (E)** Right: ROIs beta modulation depth topographies averaged across subjects. No significant amplitude differences were found.

Targets at three distances (4, 7 and 10 cm) and eight directions (45° separation) appeared on a screen in unpredictable order at 3-s interval. Targets were displayed as circles with radius varying according to distance (short: 0.5 cm; medium: 0.88 cm; long: 1.25 cm; Figure 1A). Subjects had to reach the target as soon as possible by moving a cursor from the central point with out-and-back movements sharply reversing within the target without stopping. Also, movements had to be accurate and fast with overlapping strokes without corrections. The cursor position on the screen was always visible; targets turned gray when hit. After a training where they reached 95% hit rate, subjects performed a 96-movement block.

As in previous publications (Ghilardi et al., 2000, 2003; Perfetti et al., 2011), several measures were computed for each movement. Here we report: reaction time (time from target appearance to movement onset); movement time (duration of the outgoing movement); movement extent (length of the segment from onset to reversal); and amplitude of peak velocity, which reflects the force used to reach the target. Movements with parameters outside two SD were excluded from analyses.

### EEG Recording and Data Preprocessing

High-density EEG data were acquired with Net Amp 300 amplifier (250 Hz sampling rate, online reference: Cz) and Net Station 5.0 software. Impedances were maintained below 50 kΩ throughout the recording. Data were preprocessed using EEGLAB v13.6.5b toolbox (Delorme and Makeig, 2004; Makeig et al., 2004). EEG preprocessing is detailed in the Supplementary Material. After preprocessing, epochs were time-locked to movement onset, resulting in 3.5 s epochs (−1 to 2.5 s).

Time-frequency representations were computed within beta range (15–30 Hz) using Complex Morlet Wavelets at linearly spaced frequencies (0.5 Hz bins, 10 cycles). Data were normalized on the average of beta power over the entire epoch. We computed the peak-to-peak difference (ERS-ERD, modulation depth) over the left and right sensorimotor areas (17 electrodes/area, Figure 1B). For each participant, two personalized regions of interest (ROIs) were defined by the electrode with the maximum modulation depth and the six neighbors (Figure 1C, Supplementary Figure S1). In one subject no beta modulation was observed over the right ROI. Time-frequency representations were re-computed on the left and right ROIs (1:55 Hz, 0.5 Hz bins, 3:10 wavelet cycles). After normalization by total power, beta ERS, ERD, and modulation depth magnitude, as well as peak timing values, were extracted for statistical analysis.

### Statistical Analyses

SPSS-based repeated measures one-way analyses of variance (rm-ANOVAs) were run on performance indices, beta ERS, ERD and modulation depth with target distance (Short, Medium, Long) as factor. Violations of sphericity assumption were Greenhouse-Geisser-corrected; significant main effects (*p* < 0.05) were followed by Bonferroni-corrected pairwise comparisons. JASP-based Bayes factor (BF) analysis was further applied to assess the validity of our results (see Supplementary Material). Finally, we characterized specific contributions of peak velocity and movement time on movement extent with single-subject multiple regression analysis. Possible associations between beta modulation and performance indices were assessed with Spearman rank correlation analysis.

## Results

### Movement Extent Results From Scaling Force to the Appropriate Target Extent

**Figure 2:**
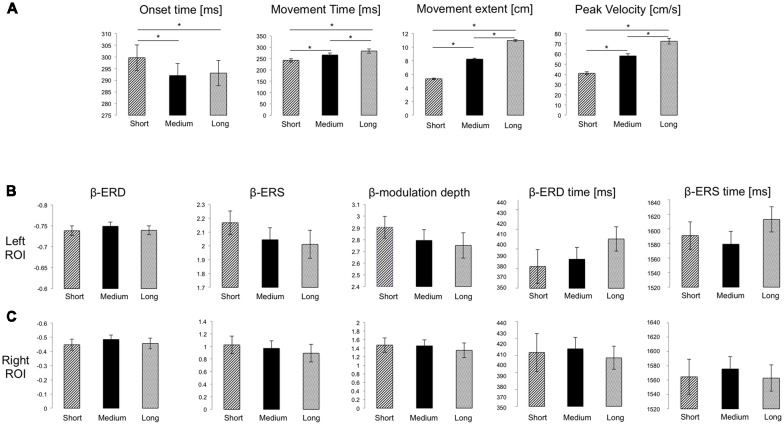
**(A)** Mean and SE of performance measures for the three target distances. Significant Bonferroni-corrected *post hoc* pairwise comparisons (*p* < 0.05) are marked with *. **(B,C)** Mean and SE of the magnitude of beta event-related desynchronization (ERD), event-relatedsynchronization (ERS) and modulation depth (dimensionless), as well as of ERD and ERS timing for the Left and Right ROIs, respectively. No significant differences were found.

All participants completed the task without difficulty or fatigue. Movements were overall straight with bell-shaped velocity profiles, with significantly different extents, and reached on average the appropriate target distance (*F*_(1.226, 41.697)_ = 2954.04, *p* < 0.001, $\eta_{\text{p}}^{2}$ = 0.99, Figure 2A). Importantly, both average peak velocity and movement time increased significantly with target distance (*F*_(1.067, 36.27)_ = 375.55, *p* < 0.001, $\eta_{\text{p}}^{2}$ = 0.92; *F*_(1.437, 48.87)_ = 141.5, *p* < 0.001, $\eta_{\text{p}}^{2}$ = 0.84, respectively; Figure 2A), a result confirmed by Bayesian rm-ANOVA (see Supplementary Table S1).

To ascertain the relative contribution of peak velocity and movement time, we performed a multiple regression on each subject’s data. The combination of peak velocity and movement time explained on average more than 90% of movement extent variance (*R*^2^ mean ± SD: 0.93 ± 0.06; range: 0.69–0.99). In all subjects, the major contributor of the variance in movement extent was variation of peak velocity (standardized coefficient Beta, mean ± SD: 0.86 ± 0.06; range: 0.73–1.02), while variation of movement time played a lesser role (standardized coefficient Beta, mean ± SD: 0.39 ± 0.09; range: 0.12–0.61). Nevertheless, both contributions were statistically significant in all subjects. Altogether, these findings suggest that movement extent mostly resulted from planning of a force appropriately scaled to the target extent with minimal adjustments of movement duration.

### Movement Extent Does Not Affect Beta Modulation Magnitude

We then determined whether target distance affects the magnitude of movement-related beta ERD, ERS and modulation depth. We first focused on the left ROI, where beta modulation depth was greater (mean ± SD; left ROI: 2.83 ± 0.53; right ROI: 2.34 ± 0.59; two-tailed *t*-test: *t*_(33)_ = 3.67, *p* = 0.001).

As in previous reports (Nelson et al., 2017), average modulation depth was higher for the last 16 movements (mean ± SD: 2.914 ± 0.73) than for the first 16 (2.635 ± 0.50, two-tailed *t*-test: *t*_(34)_ = 2.74 *p* = 0.0097). However, we found no significant effect of target distance on ERD (*F*_(2, 68)_ = 2.37, *p* = 0.101, $\eta_{\text{p}}^{2}$ = 0.065), ERS (*F*_(1.67, 56.69)_ = 2.46, *p* = 0.104, $\eta_{\text{p}}^{2}$ = 0.067) and modulation depth magnitude (*F*_(1.67, 57.61)_ = 2.34, *p* = 0.109, $\eta_{\text{p}}^{2}$ = 0.066; Figures 1D, 2B). Also, peak ERD and ERS timings were similar for the three target distances (ERD: *F*_(2, 68)_ = 1.46, *p* = 0.24, $\eta_{\text{p}}^{2}$ = 0.04; ERS: *F*_(2, 68)_ = 1.87, *p* = 0.162, $\eta_{\text{p}}^{2}$ = 0.05).

Analogous results were found for the right ROI (Figures 1E, 2C): target distance did not affect the magnitude of ERD (*F*_(2, 66)_ = 2.1, *p* = 0.131, $\eta_{\text{p}}^{2}$ = 0.06), ERS (*F*_(2, 66)_ = 1.81, *p* = 0.17, $\eta_{\text{p}}^{2}$ = 0.05) and modulation depth (*F*_(2, 68)_ = 1.86, *p* = 0.16, $\eta_{\text{p}}^{2}$ = 0.05) as well as the ERS (*F*_(1.49, 49.03)_ = 0.18, *p* = 0.77, $\eta_{\text{p}}^{2}$ = 0.005) and ERD (*F*_(1.67, 54.94)_ = 0.18, *p* = 0.79, $\eta_{\text{p}}^{2}$ = 0.005) peak timings. These results were confirmed with Bayesian statistics (see Supplementary Table S2).

Finally, there were no significant correlation between EEG parameters and performance indices (Spearman rank correlation: range *r_s_* = −0.287−0.308 to *p* > 0.05).

## Discussion

The main result of this study is that the magnitudes of beta ERD, ERS and modulation depth over the sensorimotor areas do not change with either movement length or target direction (see Supplementary Table S3 and Supplementary Figure S2). Also, we found no significant effect of target distance on ERD and ERS timing on both ROIs. Finally, in agreement with previous results, beta modulation depth increased significantly from the beginning to the end of practice (Nelson et al., 2017).

The movements produced with this reaching task had bell-shape velocity profiles that were appropriately scaled to the target distance. Indeed, peak velocity almost doubled for the long target compared to the short, while movement time increased only by 40 ms (less than 20% increase). This suggests that, as previously reported (Gordon et al., 1994a,b), movement extent in this task mainly resulted from planning a force that was appropriate to the distance. This may not be the case when using other paradigms with 3-D movements and greater distances (Ferraina et al., 2009; Hadjidimitrakis et al., 2015; Bosco et al., 2016) or context changes (Mirabella et al., 2008). Nevertheless, our results suggest that movement-related beta oscillatory activity is not significantly affected by force. This conclusion is in accordance with some previous results but contradicts others. In particular, Stančák et al. (1997) found that, when external loads opposed finger extension, beta ERS amplitude increased with higher loads. One explanation for this result is that the applied load might have influenced the proprioceptive drive: this, in turn, might have enhanced post-movement reactivation of sensory areas resulting in a greater ERS magnitude. A similar mechanism could also explain the findings of higher rate finger extensions linked to greater beta ERS amplitude (Parkes et al., 2006); as movement onsets and offsets were controlled by finger contacts with a button, the blocks with faster (more frequent) movements might have resulted in greater somatosensory input. Finally, a study showed a positive relationship between beta ERS amplitude and force output in isometric wrist flexion movements (Fry et al., 2016). While muscle pattern and proprioceptive feedback did not change, force targets were presented in ascending order and a trial-by-trial normalization was applied. Hence, task design and baseline choice do not allow distinguishing the effect of force from that of practice. Indeed, a similar study with isometric elbow flexion, where the required force level was randomized and each trial was normalized by the total power of all the trials, found no relationship between force level and beta ERS magnitude. In addition, as shown previously (Moisello et al., 2015; Nelson et al., 2017) and here, beta ERS and modulation depth steadily increase with practice, an effect that is not related to the overall increase of mean power and has no correlation with performance changes. Therefore, it is possible that changes of ERS and beta modulation reflect plasticity-related mechanisms.

## Data Availability

All datasets generated for this study are included in the manuscript and/or the supplementary files. The data that support the findings of this study are available from the corresponding author (ET) upon reasonable request.

## Author Contributions

MG: study conception and design. ET, SR, RM, AN and NL: acquisition. ET, MG, RM, NL and SG: analyses. ET, MG, SR: interpretation of the results. ET and MG: manuscript drafting and revising. All the authors agreed to be accountable for all aspects of the work in ensuring that questions related to the accuracy or integrity of any part of the work are appropriately investigated and resolved.

## Conflict of Interest Statement

The authors declare that the research was conducted in the absence of any commercial or financial relationships that could be construed as a potential conflict of interest.
