# Supplementary material for: Beta Modulation Depth Is Not Linked to Movement Features
Source: Front Behav Neurosci. 2019 Mar 14;13:49. doi: 10.3389/fnbeh.2019.00049 (PMC6426772; doi:10.3389/fnbeh.2019.00049)
Supplement: Supplementary file 1 [file Table_1.pdf]

Table 1. Results of Bayesian repeated measure ANOVAs on movement extent, movement time, peak velocity and reaction time with Target distance as factor.

Movement extent

Model Comparison

Models	P(M)	P(M|data)	BF_M	BF_{10}	error %
Null model (incl. subject)	0.5	2.786e -73	2.786e -73	1	
RM Factor Target distance	0.5	1	3.589e +72	3.589e +72	1.129

Post Hoc Comparisons - Target distance

		Prior Odds	Posterior Odds	$BF_{10, U}$	error %
Short	Medium	0.587	5.901e +30	1.005e +31	2.863e -33
	Long	0.587	9.256e +31	1.576e +32	1.272e -34
Medium	Long	0.587	1.421e +29	2.419e +29	5.522e -32

Movement time

Model Comparison

Models	P(M)	P(M|data)	BF_M	BF_{10}	error %
Null model (incl. subject)	0.5	4.186e -22	4.186e -22	1	
RM Factor Target distance	0.5	1	2.389e +21	2.389e +21	0.616

Post Hoc Comparisons - Target distance

		Prior Odds	Posterior Odds	$BF_{10, U}$	error %
Short	Medium	0.587	5.003e +8	8.517e +8	1.223e -12
	Long	0.587	8.564e +11	1.458e +12	2.590e -16
Medium	Long	0.587	5.345e +8	9.099e +8	1.106e -12

Reaction time

Model Comparison

Models	P(M)	P(M|data)	BF $_M$	BF $_{10}$	error %
Null model (incl. subject)	0.5	5.182e -5	5.182e -5	1	
RM Factor Target distance	0.5	1	19296.81	19296.81	1.362

Post Hoc Comparisons - Target distance

		Prior Odds	Posterior Odds	BF $_{10, U}$	error %
Short	Medium	0.587	4811.046	8190.394	5.860e -7
	Long	0.587	305.934	520.826	3.744e -8
Medium	Long	0.587	0.135	0.231	6.884e -7

Peak velocity

Model Comparison

Models	P(M)	P(M|data)	BF $_M$	BF $_{10}$	error %
Null model (incl. subject)	0.5	1.338e -34	1.338e -34	1	
RM Factor Target distance	0.5	1	7.476e +33	7.476e +33	1.183

Post Hoc Comparisons - Target distance

		Prior Odds	Posterior Odds	BF $_{10, U}$	error %
Short	Medium	0.587	9.940e +16	1.692e +17	5.131e -22
	Long	0.587	6.731e +16	1.146e +17	8.064e -22
Medium	Long	0.587	2.127e +15	3.620e +15	8.267e -20

Note. The posterior odds have been corrected for multiple testing by fixing to 0.5 the prior probability that the null hypothesis holds across all comparisons (Westfall, Johnson, & Utts, 1997). Individual comparisons are based on the default t-test with a Cauchy (0, r = 1/sqrt(2)) prior. The "U" in the Bayes factor denotes that it is uncorrected.
