# Supplementary material for: Beta Modulation Depth Is Not Linked to Movement Features
Source: Front Behav Neurosci. 2019 Mar 14;13:49. doi: 10.3389/fnbeh.2019.00049 (PMC6426772; doi:10.3389/fnbeh.2019.00049)
Supplement: Supplementary file 2 [file Table_2.pdf]

Table 2. Results of Bayesian repeated measure ANOVAs on Left and Right peak Beta ERD, ERS, modulation depth and peak ERD and ERS timing with Target distance as factor.

Left ROI Beta modulation

Model Comparison

Models	P(M)	P(M|data)	BF_M	BF_{10}	error %
Null model (incl. subject)	0.5	0.631	1.708	1	
RM Factor Target distance	0.5	0.369	0.586	0.586	0.963

Left ROI ERD

Model Comparison

Models	P(M)	P(M|data)	BF_M	BF_{10}	error %
Null model (incl. subject)	0.5	0.635	1.736	1	
RM Factor Target distance	0.5	0.365	0.576	0.576	0.871

Left ROI ERS

Model Comparison

Models	P(M)	P(M|data)	BF_M	BF_{10}	error %
Null model (incl. subject)	0.5	0.632	1.718	1	
RM Factor Target distance	0.5	0.368	0.582	0.582	0.887

Left ROI Peak Timing ERS

Model Comparison

Models	P(M)	P(M|data)	BF_M	BF_{10}	error %
Null model (incl. subject)	0.5	0.717	2.538	1	
RM Factor Target distance	0.5	0.283	0.394	0.394	1.001

Left ROI Peak Timing ERD

Model Comparison

Models	P(M)	P(M|data)	BF_M	BF_{10}	error %
Null model (incl. subject)	0.5	0.775	3.435	1	
RM Factor Target distance	0.5	0.225	0.291	0.291	0.626

Right ROI ERD

Model Comparison

Models	P(M)	P(M|data)	BF_M	BF_{10}	error %
Null model (incl. subject)	0.5	0.677	2.096	1	
RM Factor Target distance	0.5	0.323	0.477	0.477	1.24

Right ROI ERS

Model Comparison

Models	P(M)	P(M|data)	BF_M	BF_{10}	error %
Null model (incl. subject)	0.5	0.716	2.516	1	
RM Factor Target distance	0.5	0.284	0.397	0.397	3.112

Right ROI Beta modulation

Model Comparison

Models	P(M)	P(M|data)	BF_M	BF_{10}	error %
Null model (incl. subject)	0.5	0.722	2.593	1	
RM Factor Target distance	0.5	0.278	0.386	0.386	0.556

Right ROI Peak Timing ERD

Model Comparison

Models	P(M)	P(M|data)	BF_M	BF_{10}	error %
Null model (incl. subject)	0.5	0.907	9.761	1	
RM Factor Target distance	0.5	0.093	0.102	0.102	0.675

Right ROI Peak Timing ERS

Model Comparison

Models	P(M)	P(M|data)	BF_M	BF_{10}	error %
Null model (incl. subject)	0.5	0.906	9.679	1	
RM Factor Target distance	0.5	0.094	0.103	0.103	1.044
