# Supplementary material for: Beta Modulation Depth Is Not Linked to Movement Features
Source: Front Behav Neurosci. 2019 Mar 14;13:49. doi: 10.3389/fnbeh.2019.00049 (PMC6426772; doi:10.3389/fnbeh.2019.00049)
Supplement: Supplementary file 3 [file Table_3.pdf]

Table 3. Results of Bayesian repeated measure ANOVAs on Left and Right peak Beta ERD, ERS, modulation depth and peak ERD and ERS timing with Target direction as factor.

Left ROI ERD

Model Comparison

Models	P(M)	P(M|data)	BF_M	BF_{10}	error %
Null model (incl. subject)	0.5	0.972	34.824	1	
RM Factor Target direction	0.5	0.028	0.029	0.029	0.33

Left ROI ERS

Model Comparison

Models	P(M)	P(M|data)	BF_M	BF_{10}	error %
Null model (incl. subject)	0.5	0.972	34.095	1	
RM Factor Target direction	0.5	0.028	0.029	0.029	0.415

Left ROI Beta modulation depth

Model Comparison

Models	P(M)	P(M|data)	BF_M	BF_{10}	error %
Null model (incl. subject)	0.5	0.99	97.041	1	
RM Factor Target direction	0.5	0.01	0.01	0.01	0.27

Right ROI ERD Target direction

Model Comparison

Models	P(M)	P(M|data)	BF_M	BF_{10}	error %
Null model (incl. subject)	0.5	0.957	22.188	1	
RM Factor Target direction	0.5	0.043	0.045	0.045	0.351

Right ROI ERS

Model Comparison

Models	P(M)	P(M|data)	BF_M	BF_{10}	error %
Null model (incl. subject)	0.5	0.986	71.571	1	
RM Factor Target direction	0.5	0.014	0.014	0.014	0.418

Right ROI Beta modulation depth

Model Comparison

Models	P(M)	P(M|data)	BF_M	BF_{10}	error %
Null model (incl. subject)	0.5	0.99	95.663	1	
RM Factor Target direction	0.5	0.01	0.01	0.01	0.501
