# Supplementary material for: Beta Modulation Depth Is Not Linked to Movement Features
Source: Front Behav Neurosci. 2019 Mar 14;13:49. doi: 10.3389/fnbeh.2019.00049 (PMC6426772; doi:10.3389/fnbeh.2019.00049)
Supplement: Supplementary file 4 [file Presentation_1.pdf]

Supplemental material

EEG Data Preprocessing

The continuous signal was first FIR filtered (1-80 Hz, 60 Hz Notch filtered), segmented in 4-s epochs centered on target onset (96 epochs) and visually inspected to remove sporadic artifacts and channels with bad signal. Stereotypical artifacts, such as eye movements and heartbeat, were removed using Independent Component Analysis with Principal Component Analysis-based dimension reduction (max 108 ICs). Spherical spline interpolation was applied to reconstruct removed bad electrodes; those located on the cheeks and neck were removed, resulting in a total of 180 electrodes. Re-reference to overall signal average was finally applied.
Following preprocessing, epochs representing invalid movements were rejected. After trial rejection, the average (±SD) number of trials per subject was 77.85±9.42 (Short: 26.26±3.73; Medium: 26.5±3.84; Long: 25.09±4.90).

EEG analysis on target direction

Beta ERD, ERS and modulation depth values were extracted for each direction of the targets on the screen. (0°, 45°, 90°, 135°, 180°, 225°, 270°, 315°; mean trials: 10.05±1.43; Supplemental Figure 2). Three repeated measure ANOVA were run to test for differences in beta magnitude. Results showed no significant effect of target direction on ERD, ERS and beta modulation depth [ERD: $F(3.487, 11.552) = 0.978$, $p = .415$, $\eta 2p = 0.028$; ERS: $F(7, 238) = 0.982$, $p = .445$, $\eta 2p = 0.028$; modulation depth: $F(5.089, 173.027) = 0.547$, $p = .743$, $\eta 2p = 0.016$).

Behavioral and EEG Bayesian statistics

To confirm the validity of our results, we run Bayesian repeated measure ANOVAs with JASP software (Version 9.0) and computed the Bayes Factor (BF), a statistical index that quantifies the probability of the data under the null hypothesis model (H_0) compared to the alternative one (H_1). Results of Bayesian statistics for behavioral and EEG data are reported in Supplemental Tables 1, 2 and 3. For what concerns the behavioral data, all the alternative models (existence of a difference among target distances) outperform the null one (Table 1). BF analysis of ERS, ERD and beta modulation depth support our results with frequentist approach, as the alternative model on average predicts the observed data less than 0.6 times better than the null one ($BF_{10}=1$) (Supplemental Tables 2 and 3).

Supplemental figure legend

Supplemental Figure 1. Topographies showing selected Left and Right ROIs for each subject.

Supplemental Figure 2. Mean and SE of the magnitude of Left and Right beta ERD, ERS and modulation depth (dimensionless) for each target direction.
