# Supplementary figures and images for: Beta Modulation Depth Is Not Linked to Movement Features
Source: Front Behav Neurosci. 2019 Mar 14;13:49. doi: 10.3389/fnbeh.2019.00049 (PMC6426772; doi:10.3389/fnbeh.2019.00049)

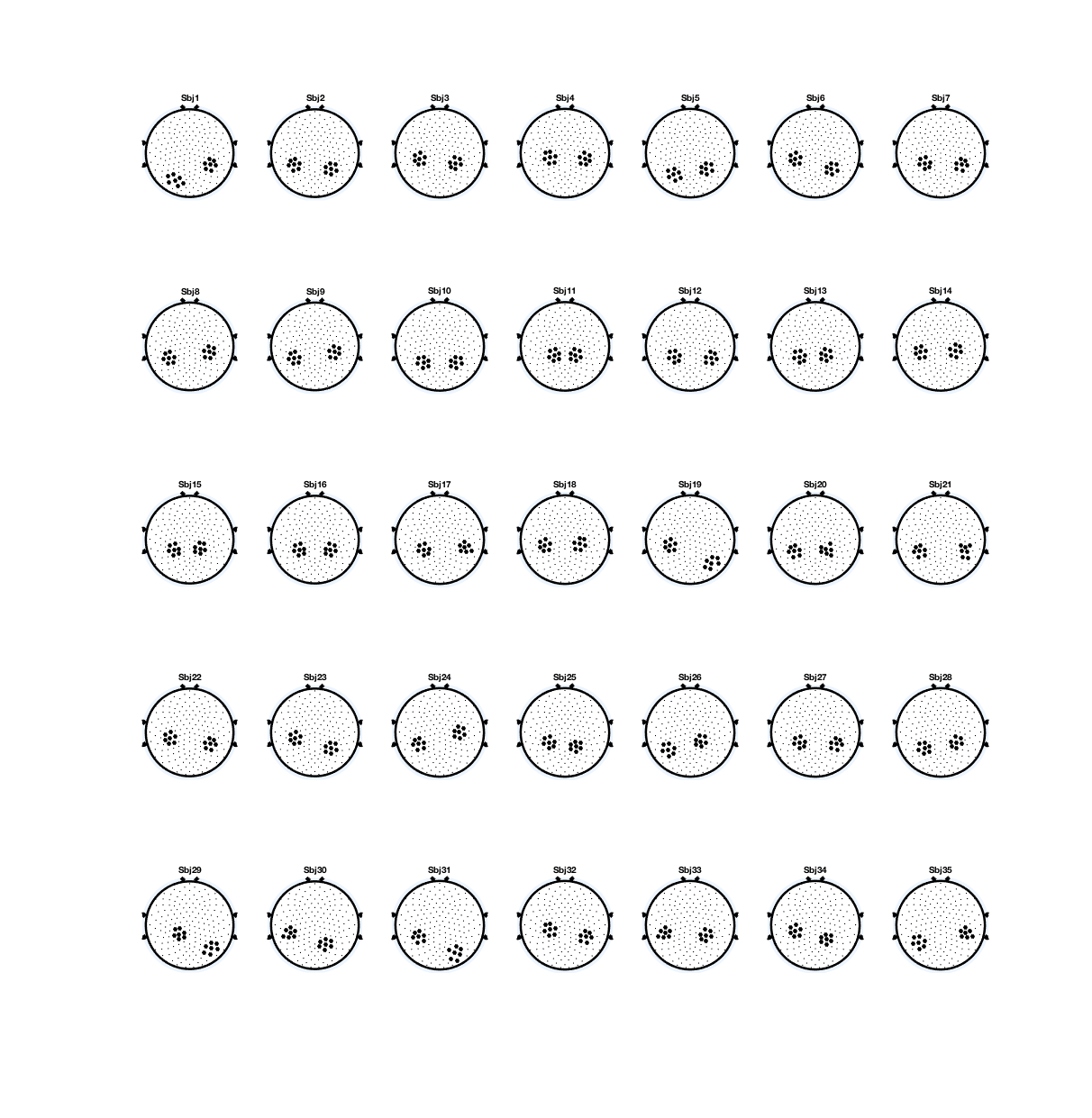

Supplement: Supplementary file 5 [file Image_1.TIF]
